# The Role of microRNAs and Long Non-Coding RNAs in the Regulation of the Immune Response to *Mycobacterium tuberculosis* Infection

**DOI:** 10.3389/fimmu.2021.687962

**Published:** 2021-06-24

**Authors:** Manikuntala Kundu, Joyoti Basu

**Affiliations:** Department of Chemistry, Bose Institute, Kolkata, India

**Keywords:** *Mycobacterium tuberculosis*, microRNAs, long non-coding RNAs, immune response, autophagy, apoptosis, inflammation

## Abstract

Non-coding RNAs have emerged as critical regulators of the immune response to infection. MicroRNAs (miRNAs) are small non-coding RNAs which regulate host defense mechanisms against viruses, bacteria and fungi. They are involved in the delicate interplay between *Mycobacterium tuberculosis*, the causative agent of tuberculosis (TB), and its host, which dictates the course of infection. Differential expression of miRNAs upon infection with *M. tuberculosis*, regulates host signaling pathways linked to inflammation, autophagy, apoptosis and polarization of macrophages. Experimental evidence suggests that virulent *M. tuberculosis* often utilize host miRNAs to promote pathogenicity by restricting host-mediated antibacterial signaling pathways. At the same time, host- induced miRNAs augment antibacterial processes such as autophagy, to limit bacterial proliferation. Targeting miRNAs is an emerging option for host-directed therapies. Recent studies have explored the role of long non-coding RNA (lncRNAs) in the regulation of the host response to mycobacterial infection. Among other functions, lncRNAs interact with chromatin remodelers to regulate gene expression and also function as miRNA sponges. In this review we attempt to summarize recent literature on how miRNAs and lncRNAs are differentially expressed during the course of *M. tuberculosis* infection, and how they influence the outcome of infection. We also discuss the potential use of non-coding RNAs as biomarkers of active and latent tuberculosis. Comprehensive understanding of the role of these non-coding RNAs is the first step towards developing RNA-based therapeutics and diagnostic tools for the treatment of TB.

## Introduction

Tuberculosis (TB) is a global health problem. About one third of the world’s population is infected with *Mycobacterium tuberculosis*, the causative agent of TB. Of these, about 5 to 10% of infected individuals develop active disease. Around 10 million new TB infections and 1.4 million deaths were reported in 2019 (1). In latent TB (LTBI), *M. tuberculosis* remains walled off within a granuloma in the lung for long periods of time in a dormant state with no symptoms of disease and without a marked immune response. The bacilli then reactivate under favorable conditions, most notably when the host is immunocompromised as in the case of HIV infection. A reduction in the global burden of TB, requires a means of diagnosing as well as treating latent TB. The problems of TB are further exacerbated by the growing increase in multidrug-resistant (MDR), extensively drug-resistant (XDR) and totally drug resistant TB. With this background, it is evident that there is an increasing need to develop newer approaches towards therapy of TB. Host-directed therapies (HDTs) provide the option of manipulating the host immune response to thwart disease, without the development of drug-resistant bacilli. Effective HDTs require a detailed knowledge of the immune response to infection.

MicroRNAs (miRNAs) are non-coding RNAs which are conserved across species and phyla and are typically 20 to 22 nucleotides in length (2). In recent years it has become evident that miRNAs regulate the interactions between hosts and pathogens (3–5). They play important roles in regulating the immune response to bacterial pathogens such as *Listeria monocytogenes*, *Helicobacter pylori*, *Pseudomonas aeruginosa*, *Salmonella typhimurium* and *M. tuberculosis* (5). miRNAs regulate the response of innate immune cells such as macrophages, to infection. Macrophages are the sentinels of the host immune defense system. One of the first steps of bacterial infection is the sensing of the pathogen associated molecular patterns (PAMPs) by host pattern recognition receptors (PRRs) expressed on macrophages. This triggers a cascade of signaling events which culminate in reprogramming of the host transcriptome so that the bacterium is effectively thwarted. The bacterium responds by remodeling its own transcriptome to adjust metabolism and to express virulence genes. Bacteria also hijack the innate immune pathways of the host for their own benefit (6). In the case of *M. tuberculosis* infection, the host-pathogen interaction impacts processes such as apoptosis, autophagy, cytokine production, macrophage polarization and MHC class II expression. Recent studies have shown that many of these processes are regulated by miRNAs (7–9). Long noncoding RNAs (lncRNAs) are transcripts that are longer than 200 nucleotides, but do not code for proteins (10–12). LncRNAs interact with proteins such as chromatin remodelers and are regulators of innate immunity (13, 14). Differential regulation of lncRNAs modulates the response of immune cells to mycobacterial infection (15, 16).

In addition to the potential use of miRNA or lncRNA-targeted drugs in HDT, several studies have explored the potential of miRNAs (17) and lncRNAs (18) as biomarkers for active TB as well as LTBI, thus underscoring the translational potential of miRNAs as markers of disease. Here, we will focus on regulation of the macrophage immune response to *M. tuberculosis* infection by miRNAs and lncRNAs, and touch upon their potential as biomarkers of disease. We will restrict our discussion primarily to how these ncRNAs rewire the innate immune response to regulate inflammation, apoptosis, macrophage polarization and autophagy, which determine the outcome of *M. tuberculosis* infection. The immune responses to other pathogenic mycobacteria, have not been discussed in detail in this review.

## Biogenesis of miRNAs

miRBase, an online repository of miRNAs, lists 4885 mature miRNAs in 271 species (release 22, March 2018). miRNA genes are transcribed by RNA polymerase II, as 5’-capped and 3’-polyadenylated precursors of 200 to 300 nucleotides (19). They are processed by nuclear RNase Drosha and the RNA binding protein DiGeorge syndrome critical region 8 (DGCR8) into 60-70 nucleotide premiRNAs with hairpin structures and exported to the cytoplasm with the help of Exportin 5 and RAN GTPase. In the cytoplasm, the pre-miRNA is cleaved by the RNase III Dicer in combination with the HIV TAR RNA‐binding protein (TRBP) or Protein Activator of PKR (PACT) RNA-binding proteins into 16-24 bp double stranded RNA. The guide RNA strand then associates with a protein of the Argonaute (AGO) family while the passenger strand is degraded. The AGO-associated miRNA strand is a part of the RNA-induced silencing complex (RISC). It interacts with mRNA *via* base pairing. The AGO proteins along with other partners recruit other players such as the deadenylase complexes PAN2-PAN3 and CCR4-NOT to repress translation and promote degradation of target mRNAs (20). miRNA biogenesis is regulated at the post-translational level through modifications such as phosphorylation and sumoylation (19).

## Mammalian Toll-Like Receptors and RLRs

MiRNAs regulate innate immune responses by targeting inflammatory pathways. PRRs recognize PAMPS to trigger inflammatory signaling. The role of Toll-like receptors (TLRs) has been extensively studied in the context of *M. tuberculosis* infection (21). The cytosolic sensors cyclic GMP-AMP synthase (cGAS), interferon-activable protein 204 (IFI204), and absent in melanoma 2 (AIM2) recognize *M. tuberculosis* DNA, while retinoic acid-inducible gene-I (RIG-I), melanoma differentiation‐associated protein 5 (MDA5), and protein kinase R (PKR) detect RNA (22). Nucleotide‐binding oligomerization domain (NOD) 2 recognizes muramyl dipeptide and regulates inflammatory cytokine production during *M. tuberculosis* infection (23).

The best studied pathway inhibited by miRNAs involves signaling through TLRs. TLRs are expressed on the plasma membrane [TLRS 1, 2, 5, 6; and 11 (expressed in mice but not in humans)] or on endosomes [TLRs 3, 7-8, 9; and 13 (expressed in mice but not in humans)] (24). Each TLR (or a combination of TLRs) senses PAMPs such as tricayllipopeptides (TLR1/2), diacyllipopeptides (TLR2/TLR6), lipopolysaccharide (LPS) (TLR4), flagellin (TLR5), dsRNA (TLR3), ssRNA (TLRs 7 and 8) or CpG DNA and hemozoin (TLR9). Once a ligand binds to the extracellular domain of a TLR, the intracellular Toll-IL-1-resistance (TIR) domains dimerize and recruit adaptor proteins such as myeloid differentiating factor 88 (MyD88) and TIR domain-containing adaptor inducing interferon-β (TRIF), followed by the sequential recruitment and activation of IL-1R-associated kinase (IRAK)-4, IRAK-1 and IRAK-2. Subsequently, there is engagement of downstream adaptor molecules such as TNF receptor-associated factors (TRAFs), which then undergo K63-linked ubiquitination to activate the IκB kinase (IKK) complex (25). The IKK complex phosphorylates IκB-α, triggering its ubiquitination and proteasomal degradation, release of nuclear factor κ-light-chain-enhancer of activated B cells (NF-κB) p65, its translocation to the nucleus, and regulation of gene transcription, including those associated with inflammation (26). TLR3- or TLR4-mediated TRIF-dependent signaling leads to activation of the non-canonical IKKs Tank-binding protein kinase 1 (TBK1) and IKKε, and the phosphorylation and nuclear translocation of IFN regulatory factor (IRF) 3. miRNAs limit inflammation by targeting intermediates in this pathway.

RIG-I receptors (RLRs) are RNA sensors localized in the cytosol (27–29). There are three members, RIG-I, MDA5 and laboratory of genetics and physiology 2 (LGP2). RIG-I and MDA5 have two amino-terminal caspase activation and recruitment domains (CARDs), which mediate downstream signal transduction. RIG-I and MDA5 are essential for antiviral defense and type I interferon induction (30, 31). Interaction of RIG-I with mitochondrial anti-viral signaling protein (MAVS) is followed by activation of TBK1 and IKKϵ, which in turn activate IRF3 and IRF7 (32).

## miRNAs Target the Innate Immune Response During *M. tuberculosis* Infection

Phagocytosis and maturation of the phagosome are key steps in the clearance of bacterial pathogens by macrophages. Pathogenic mycobacteria evade maturation of the phagosome where they reside at least in part by blocking actin assembly. Early during infection of macrophages with *M. tuberculosis* there is an increase in miR-142-3p which directly targets N-Wiskott-Aldrich syndrome protein (N-WASp) to inhibit actin assembly (33). This demonstrates the miRNA-mediated regulation of phagocytosis during *M. tuberculosis* infection. MiR-146a/b, miR-155 and miR-21, form a trinity of miRNAs which regulate multiple steps of the TLR and RLR pathways (34–38) to regulate inflammation. These miRNAs are regulated upon activation of TLRs. miR-21 upregulation during *Mycobacterium leprae* infection is a bacterial strategy to escape the vitamin D-dependent induction of antimicrobial peptides (39). Downregulation of let-7 family members is also characteristic of the response of macrophages to bacterial infection (40, 41). Phosphatidylinositol-3,4,5-trisphosphate 5-phosphatase (SHIP1) is a negative regulator of NF-κB signaling. miR-155 targets SHIP1 in *M. tuberculosis*-infected macrophages (42), leading to the activation of the serine/threonine kinase Akt, likely facilitating the survival of *M. tuberculosis* in macrophages. miR-155 also targets BTB and CNC homology 1 (Bach1), a transcriptional repressor of heme oxygenase-1 (HO-1) and inhibits expression of interleukin-6 (*Il6*) to emerge as a regulator of the macrophage response to *M. tuberculosis*. miR-155 plays a dual role during *M. tuberculosis* infection *in vivo*. It enhances survival of macrophages as well as *M. tuberculosis*-specific T cells, providing on the one hand a niche for bacterial replication and on the other hand enabling an effective immune response (43). In support of this, miR-155^-/-^ mice control infection at the initial stages, but fail to do so after the onset of adaptive immunity. Overall, the knockout of miR-155 exacerbates infection. This has also been borne out by the studies of Iwai et al. (44) using miR-155^-/-^ mice challenged with *M. tuberculosis*. miRNA-mediated repression of the NF-κB pathway prevents exacerbated immune responses to infection. *M. tuberculosis*-induced miR-21-5p attenuates the secretion of IL-1β, IL-6 and TNF-α in RAW264.7 and THP-1 macrophages (45), whereas miR-27a directly targets IRAK4 to attenuate the production of IFN-γ, IL-β, IL-6, and TNF-α (46). There have been few studies on the regulation of miRNAs during infection of alveolar macrophages. In *M. bovis* BCG-infected alveolar macrophages, miR-124 negatively regulates inflammatory responses by directly targeting multiple components of the TLR pathways including MyD88, TRAF6 and TNF-α (47). miR-203, miR-30a and miR-149 target MyD88 in infected murine or human macrophages to downregulate proinflammatory cytokines as well as nitric oxide (NO) production (48–50). miR-20b inhibits *M. tuberculosis*-induced inflammation by directly targeting the NLRP3/casapase1/IL1β pathway (51). Further, miRNA-mediated NF-κB repression promotes the survival of *M*. *tuberculosis* in macrophages. The downregulation of let-7f during infection increases the expression of the A20 deubiquitinase. A20 targets the K63-linked ubiquitination of TRAF6 (52, 53), thereby negatively regulating the NF-κB pathway, and inhibiting inflammatory cytokine and nitric oxide production thereby facilitating the survival of *M. tuberculosis* in macrophages (40). MiRNA-125a targets TRAF6 during *M. tuberculosis* infection, to repress NF-κB (54). The TLR4/miRNA-32-5p/FSTL1 (follistin like protein 1) axis attenuates IL-1β, IL-6 and TNF-α in THP-1 and U937 cells after *M. tuberculosis* infection (55). *M. tuberculosis-*induced miR-1178 targets TLR4 and attenuates release of IFN-γ, IL-6, IL-1β, and TNF-α in human macrophages (56). miR-378d targets Rab10. It is downregulated in *M. tuberculosis-*infected THP-1 and RAW264.7 macrophages leading to enhanced production of IL-6, IL-1β and TNF-α (57). miR-206 targets tissue inhibitor of metalloproteinase 3 (TIMP3) (58). leading to increased production of MMP9 as well as inflammatory cytokines during *M*. *tuberculosis* infection. miR-99b expression is highly upregulated in infected macrophages as well as in dendritic cells. It targets TNF-α and TNFRSF-4 receptor genes to attenuate IL-12, IL-1β, IL-6 and TNF-α (59). miR-125b targets TNF-α, whereas miR-155 enhances TNF-α production by increasing TNF mRNA half-life and limiting expression of SHIP1, a negative regulator of the PI3K/Akt pathway. Rajaram et al. (60) have shown that human macrophages infected with *M. tuberculosis* induce high miR-125b expression and low miR-155 expression with correspondingly low TNF-α production. miR‐140 is upregulated in human peripheral blood mononuclear cells (PBMCs) from patients with TB. It dampens the production of IL-1β, IL-6 and TNF-α in THP-1 macrophages infected with *M. tuberculosis* (61). In *M. bovis* BCG-infected macrophages, miR-142-3p targets IRAK1 (62) and miR-146a targets IRAK-1 and TRAF-6 (63) to dampen the production of proinflammatory cytokines TNF-α, IL-6, IL-1β and the chemokine MCP-1. miR-223 targets the chemoattractants CXCL2, CCL3, and IL-6 in myeloid cells to regulate recruitment of myeloid cells to the lungs and as a result, neutrophil-driven inflammation (64). MiR-223 is upregulated in the blood and lung parenchyma of tuberculosis patients. From the above, it is evident that miRNAs play a central role in the attenuation of inflammatory responses during *M. tuberculosis* infection. **Table 1** summarizes the role of miRNAs in regulating inflammatory responses in *M. tuberculosis*-infected macrophages.

**Table 1:** MicroRNAs that target signaling in myeloid cells during *M. tuberculosis* infection to regulate phagocytosis, cytokines, chemokines, macrophage polarization and glycolysis.

MicroRNA	Target	Reference
miR-142-3p	N-Wasp	(33)
miR-155	Ship1, Bach1	(42)
miR-21-5p	Bcl-2, Tlr4	(45)
miR-27a	Irak4	(46)
miR-203, miR-30a, miR-149	Myd88	(48–50)
miR-124	Myd88, Traf6, Tnfα	(47)
miR-20b	Nlrp3	(51)
Let-7f	A20 (Tnfaip3)	(40)
miR-125a	Traf6	(54)
miR-32-5p	Fstl1	(55)
miR-1178	Tlr4	(56)
miR-378d	Rab10	(57)
miR-206	Timp3	(58)
miR-99b	Tnfα, Tnfrsf4	(59)
miR-125b	Tnfα	(60)
miR-26a-5p	Klf4	(65)
miR-132	p300	(66)
miR-20b	Nlrp3	(51)
miR-223	Cxcl2, Ccl3, Il6	(64)
miR-21	Pfk-M	(67)

Depending on the stimulus, macrophages can be directed towards distinct phenotypes in a process termed macrophage polarization. In the simplest scenario, macrophage polarization results in M1 (classically activated macrophages, proinflammatory state) or M2 (alternatively activated macrophages, anti-inflammatory state) phenotypes, a molecular event crucial for inflammation. M1 macrophages produce proinflammatory cytokines TNF-α, IL-1β, IL-12, IL-6 and IL-23 along with reactive oxygen species (ROS) and nitric oxide. They recruit other immune cells including neutrophils through the production of chemokines such as CXCL8, CCL2, CXCL11, CXCL9 and CXCL10. M2 macrophages produce anti-inflammatory cytokines such as IL-10 and TGF-β, as well as arginase which represses nitric oxide production. M2 macrophages express the mannose receptor (MR) which signals production of cytokines such as CCL17, CCL18, CCL22 and CCL24 that recruit Th2 lymphocytes, eosinophils, basophils and T regulatory (Treg) cells (68). M1 polarized macrophages efficiently clear *M. tuberculosis*, whereas M2 polarization enhances survival of the bacterium in macrophages. Virulent strains of *M. tuberculosis* drive M2 polarization (69). Macrophage polarization is influenced by miRNAs. For example, *M. tuberculosis* infection of murine macrophages, decreases miR-26a-5p, consequently derepressing the transcription factor Kruppel-like factor 4 (KLF4), favoring M2 macrophage polarization in murine macrophages (65). However, in human macrophages miR-26a and miR-132 have been reported to be upregulated (66). These target p300 to attenuate IFN*γ* responses. In contrast, miR-20b directly targets nucleotide-binding oligomerization domain, leucine rich repeat and pyrin domain containing (NLRP) 3, and its downregulation during *M. tuberculosis* infection favors M1 polarization through activation of the NLRP3/IL-1β/caspase-1 axis (51). It is evident that inflammatory responses in the macrophage can be regulated by targeting miRNAs which interfere with pathways linked to macrophage polarization.

## The Regulation of Metabolic Reprogramming by miRNAs

The phenotype of the macrophage is intimately linked to its metabolism (70). The relationship between metabolic pathways and macrophage polarization, is more complex than previously thought, However, in a broad sense, M1 macrophages rely on glycolysis (71). They present two breaks in the tricarboxylic acid (TCA) cycle which lead to accumulation of itaconate and succinate, which directly impact macrophage functions (72). Succinate stabilizes hypoxia inducible factor 1-α (HIF1-α). This activates the transcription of glycolytic genes and enhances glycolysis. M2 macrophages, on the other hand, are more dependent on oxidative phosphorylation (OXPHOS), their TCA cycle being intact. Evidence for the prevalence of glycolysis during *M. tuberculosis* infection was obtained by RNA-sequencing of the infected mouse lung tissue at 12, 18 and 30 days (73). It showed the upregulation of glycolytic genes including hexokinases (*Hk2* and *3*), phosphofructokinase (*Pfk*) family 1 and 2, glyceraldehye-3-phosphate dehydrogenase (*Gapdh*), phosphoglycerate kinase (*Pgk1*), enolase (*Eno1*), and lactate dehydrogenase A (*Ldha*); glucose transporters (*Glut1, 3* and *6*); a transporter for lactate (*Mct4*); an H^+^-ATPase involved in cytosolic pH homeostasis; and *Hif-1α* (regulatory unit of HIF-1, a transcriptional activator of several glycolytic genes which regulates inflammatory processes under normoxia (74). Treating *M. tuberculosis*-infected mice with 2-deoxyglucose depleted the highly glycolytic subset of infiltrating macrophages and increased the burden of *M. tuberculosis*. The importance of glycolysis in host defense against *M. tuberculosis* has been documented in several studies (75–78). Hackett et al. (67) have shown that persistent *M. tuberculosis* infection of macrophages is associated with miR-21-dependent negative regulation of host glycolysis. Dampening of glycolysis is mediated through targeting of phosphofructokinase muscle (PFK-M) isoform which in turn facilitates bacterial growth by limiting pro-inflammatory mediators such as IL-1β. *M. tuberculosis*-infected *MiR-21* ^−/−^ bone marrow derived macrophages (BMDMs) showed increased levels of pro-inflammatory mediators including nitric oxide synthase 2 (*Nos2*) mRNA, arginase 1 (*Arg1*), and ROS with concomitant containment of *M. tuberculosis*. Increased containment of bacteria in *MiR-21* ^−/−^ BMDMs is lost when expression of PFK-M is silenced by specific siRNA. In summary, metabolic reprogramming as a consequence of infection, can be influenced by targeting miRNA-regulated pathways. In this context, it is important to evaluate how much information obtained from experiments with mice, can be extrapolated to human infection.

## Regulation of Autophagy by miRNAs

**Figure 1:**
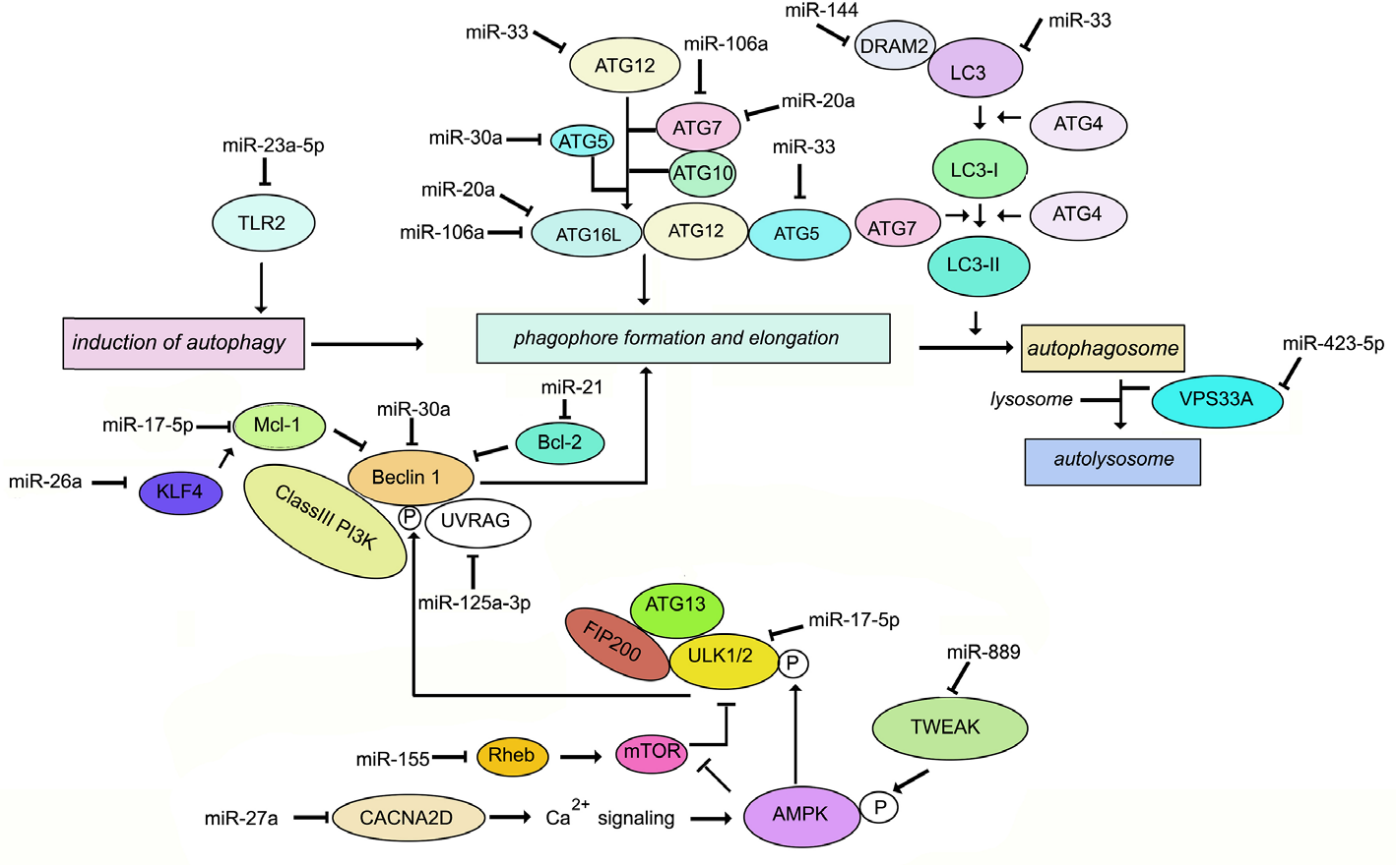
Schematic representation of the key molecules of autophagy targeted by miRNAs during *M. tuberculosis* infection.

Autophagy is regarded as a homeostatic process by which the eukaryotic cell delivers cytosolic cargo such as misfolded proteins or damaged organelles to the lysosomes for degradation. It can be classified into macroautophagy, chaperone-mediated autophagy and microautophagy. For this review, the term autophagy will refer to macroautophagy. Autophagy impacts diseases linked to inflammation such as infections, cancer, metabolic disorders, autoimmunity, neurodegeneration, cardiovascular and liver diseases (79). In brief, autophagy requires the capture of cargo destined for the lysosomes in double-membrane organelles termed autophagosomes. This occurs through the induction of a signal followed by membrane targeting, vesicle expansion and autophagosome formation. A range of signals such as starvation triggers formation of an isolation membrane usually derived from the endoplasmic reticulum (80). The Unc-51-like kinase 1 (ULK1) is released from the mammalian target of rapamycin complex 1 (mTORC1). Phosphorylated AMP-activated protein kinase (AMPK) phosphorylates ULK1 which in turn forms a complex with the 200 kDa focal adhesion kinase family-interacting protein (FIP200) and autophagy related gene (ATG) 13 to induce autophagy (81). The ULK1 complex recruits Beclin-1 (BECN1) and phosphoinositide 3-kinase regulatory subunit 4 (PIK3R4) in the phagophore membrane nucleation step. The engagement of PtdIns3-kinase class III (PtdIns3KC3) results in the production of PtdIns3P at the phagophore, recruitment of PtdIns3P binding proteins such as WD-repeat protein interacting with phosphoinositides (WIPI) 1 and WIPI2, finally leading to phagophore assembly. The tumor suppressor protein, UV-radiation resistance associated gene (UVRAG) interacts with Beclin-1 and promotes Beclin-1–PI3KC3-mediated autophagy by functioning as an adaptor (82, 83). Autophagosome elongation involves two ubiquitin-like conjugation systems, ATG12-ATG5 which is activated by ATG7 and ATG10 (E1 like and E2 like enzymes respectively) and light chain 3 (LC3 or MAP1LC3B)-phosphatidylethanolamine (PE) which is activated by ATG7 and ATG3 (E2 like enzyme) (84). ATG12-ATG5 complex directs LC3 to the target membrane where it is cleaved by the cysteine protease ATG4B, followed by conjugation of PE to the exposed glycine residue (**Figure 1**). Eventually, the autophagosomes are directed to lysosomes in a process requiring small GTPases such as Rab7 and soluble N-ethylmaleimide-sensitive factor attachment protein receptor proteins (SNAREs). In a variation of autophagy termed xenophagy, intracellular bacteria and viruses are captured with the help of autophagy adaptors for targeting to lysosomes (85, 86). Adaptors such as sequestosome 1 (SQSTM1/p62), nuclear dot protein 52 (NDP52), optineurin (OPTN), and neighbor of BRCA1 gene 1 (NBR1) form a bridge between the cargo and LC3 (87). For a more detailed understanding of autophagy and its role in the immune response to bacteria and viruses, the reader may refer to several excellent reviews (85, 86, 88–90).

Autophagy has long been recognized as a defense against *M. tuberculosis* (91–93). *M. tuberculosis* employs a range of factors to evade autophagy. For example, “enhanced intracellular survival” (eis) gene inhibits host autophagy (94). MiRNAs play a role in the regulation of autophagy during *M. tuberculosis* infection (95). Differentially regulated miR-23a-5p induction by *M. tuberculosis* inhibits autophagy in murine RAW264.7 macrophages by targeting TLR2 (96). Induction of Ca^2+^ acts as a major regulator of autophagy. Upon stimulation, entry of Ca^2+^ into the cytoplasm activates Ca^2+^/calmodulin-dependent serine/threonine kinase leading to activation of AMPK and phosphorylation of ULK1. Therefore downregulation of Ca^2+^ signaling inhibits autophagy promoting the intracellular survival of *M*. *tuberculosis* in macrophages. miR-27a targets the Ca^2+^ transporter voltage-dependent calcium channel subunit alfa2delta (CACNA2D) to limit Ca^2+^ entry into the cytoplasm and downregulate Ca^2+^ signaling (97). Mycobacterial infection induces TNF-like weak inducer of apoptosis (TWEAK) in the early phase of infection. TWEAK induces autophagy and promotes mycobacterial autophagosome maturation through activation of AMPK. *M. tuberculosis* induces MiR-889 which inhibits autophagy *via* post-transcriptional suppression of TWEAK expression to maintain mycobacterial survival in granulomas (98). The Bcl-2 family member Mcl-1 sequesters Beclin-1 to inhibit autophagy (99). *M. tuberculosis* inhibits miR17-5p and upregulates its targets Mcl-1 and STAT3, which is a transcriptional activator of Mcl-1 (100), thereby inhibiting autophagy during *M. tuberculosis* infection. miR-125a-3p regulates autophagy by targeting UVRAG (101). *M. tuberculosis* induces miR106-a in THP1 macrophages. miR-106-a could inhibit autophagy by targeting ULK1, ATG7, and ATG16L1 (102). Infection of macrophages with *M. bovis* BCG results in enhanced expression of miR-20a, which inhibits autophagy by targeting ATG7 and ATG16L1 (103). A negative correlation has been demonstrated between miR-30a expression and levels of Beclin-1 as well as ATG5 in *M. tuberculosis*-infected THP-1 cells (104). Ouimet et al. (8) have reported that miR-33 inhibits autophagy flux through repression of ATG5, ATG12 and LC3B in *M. tuberculosis-*infected macrophages. *M. tuberculosis* infection upregulates miR-144 which targets DNA damage-regulated autophagy modulator (DRAM2). DRAM2 releases Beclin-1 from the BECN1 inhibitory complex by directly interacting with Beclin1 (105). Vacuolar protein sorting 33A(VPS33A) is a direct target of miR-423-5p which is upregulated in the serum of TB patients (106). It is therefore plausible that upregulation of miR-423-5p suppresses autophagosome-lysosome fusion during active tuberculosis. Upregulation of miR-26a has been demonstrated to modulate autophagy and *M. tuberculosis* survival in human and murine macrophages by targeting Mcl-1 (65). In summary, during *M*. *tuberculosis* infection, multiple miRNAs target intermediates which are involved in autophagy. There are fewer reports of miRNA-mediated induction of autophagy during *M. tuberculosis* infection. One report is the induction of miR-155 in *M. tuberculosis*-infected macrophages, which leads to repression of the negative regulator of autophagy, RHEB, ultimately inducing autophagy and compromised bacterial survival (107). On the contrary, *M. bovis* BCG reportedly inhibits IFN*γ*-induced autophagy by induction of miR-155 and miR-31 which target the protein phosphatase 2A (PP2A) regulatory subunit, PPP2R5A (108). These differences could be due to miR-155 functioning differently in resting macrophages *vs*. IFN*γ*-activated macrophages, or due to differences in cell types infected. The role of miRNAs in the regulation of autophagy during *M. tuberculosis* infection, is summarized in **Figure 1**. Exploiting miRNA-directed therapeutics to augment autophagy, is a plausible option for the management of TB.

## Regulation of Apoptosis by miRNAs

**Figure 2:**
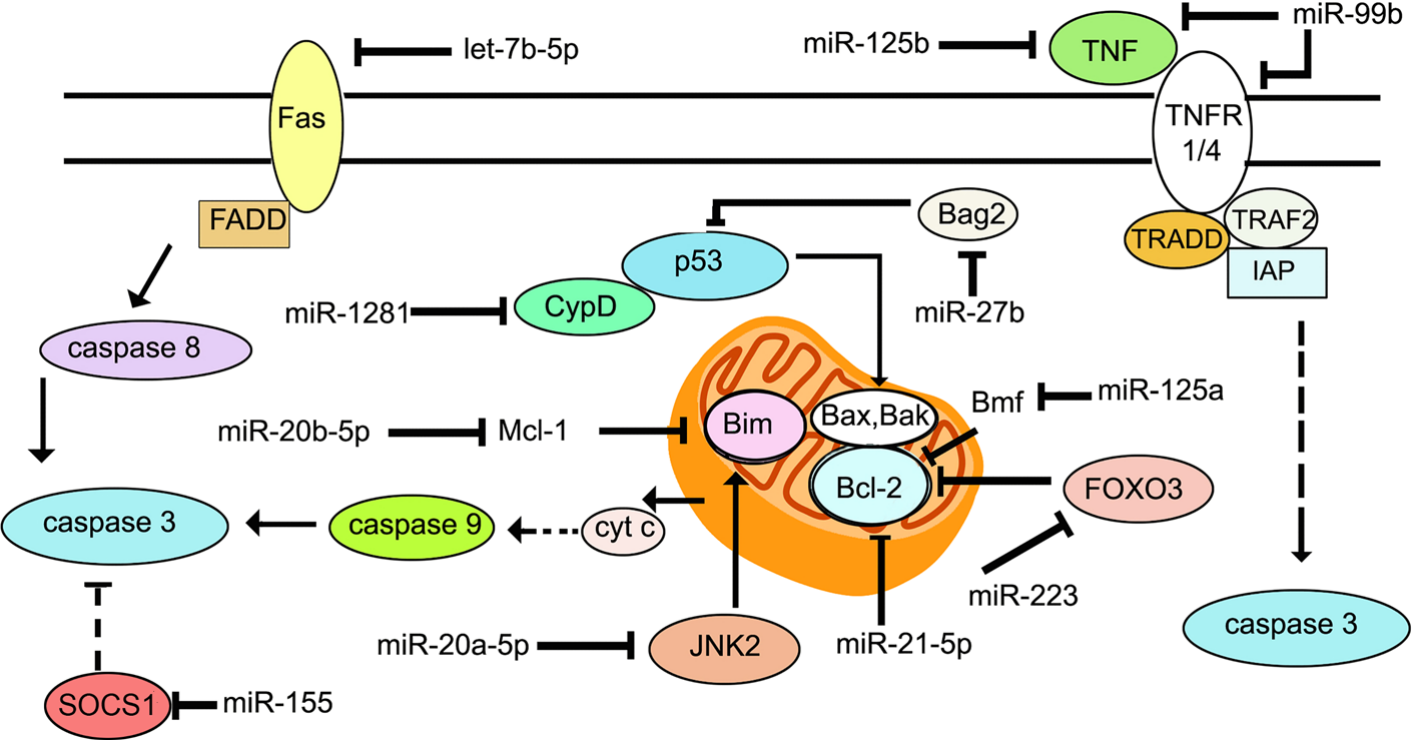
Schematic representation of the key molecules of apoptosis targeted by miRNAs during *M. tuberculosis* infection.

There are principally two major pathways leading to apoptosis that are characterized by the activation of caspases 3 and 7 (109, 110). The extrinsic apoptotic pathway involves death receptors, such as Fas and TNFR1, which are membrane-bound. Upon binding of their ligands FasL and TNFR1, they oligomerize and initiate a cascade of signaling events involving assembly of a death-inducing signaling complex (DISC) on the cytoplasmic side of the membrane. The Fas/FasL pair recruits the adaptor protein Fas-associated protein with death domain (FADD), while the TNFR1/TNF pair, recruits tumor necrosis factor receptor type 1-associated death domain protein (TRADD). This is followed by formation of the death inducing complex (DISC) which causes the self-cleavage of procaspase 8 into its active form. Caspase 8 then activates downstream caspases -3, -6 and -7. Intrinsic apoptosis occurs when the cell experiences intracellular stress such as DNA damage or oxidative stress, leading to damage of the mitochondrial outer membrane, and mitochondrial outer membrane permeability (MOMP). Cytochrome c is released into the cytosol, where it associates with the adaptor protein apoptosis protease activating factor 1 (Apaf-1) to trigger its oligomerization and formation of the apoptosome complex. The apoptosome recruits and activates caspase 9, finally leading to the activation of caspase 3. The release of cytochrome c is regulated by the Bcl-2 family of proteins that are either pro- or anti-apoptotic (111). Pro-apoptotic members such as Bax and Bak bind directly to the mitochondrial outer membrane forming pores. Anti-apoptotic members such as Mcl-1 and Bcl-2 bind and sequester the proapoptotic Bcl-2 proteins. The BH3 only proteins such as Bim, p53 upregulated modulator of apoptosis (PUMA), Bid and Bad, bind to the pro-survival Bcl-2 family proteins to sequester them (112) (**Figure 2**). Unlike apoptosis, necrotic cells undergo swelling of organelles and rupturing of cytoplasmic content into the extracellular space. The necrotic debris and damage-associated molecular pattern molecules (DAMPs) trigger inflammation and tissue damage (113). *M. tuberculosis* initiates apoptosis or necrosis in the infected host. Apoptosis protects the host and kills the bacteria through efferocytosis of the apoptotic vesicles (114). Apoptosis of infected macrophages is required for dendritic cells to acquire mycobacterial antigens for cross priming. Necrosis, on the other hand, benefits the pathogen. It releases the intracellular bacteria into the extracellular milieu to promote dissemination, and triggers inflammation. Virulent strains of *M. tuberculosis* induce necrosis (115, 116) and evade apoptosis (117). The differential regulation of apoptosis and necrosis by virulent as opposed to avirulent *M. tuberculosis* or members of the *M. tuberculosis* complex, depends at least in part, on the differential expression of miRNAs. For example, *M. bovis* BCG induces IFN*γ* by inhibiting miR-29 (118), whereas *M. tuberculosis* H37Rv upregulates miR-99b expression in murine dendritic cells and macrophages to decrease their targets TNF and TNFRSF45 (TNFR1) (59). Cytokine expression in the cells is altered to affect activation of the host immune response and the survival of intracellular mycobacteria. miR-20a-5p targets JNK2 to repress Bim expression and apoptosis in macrophages. Downregulation of miR-20a-5p in THP-1 macrophages triggers apoptosis and facilitates clearance of *M. tuberculosis* (119). miR-20b-5p on the other hand, targets Mcl-1 (120), and miR-21-5p targets Bcl-2 (45) to activate apoptosis in *M. tuberculosis*-infected macrophages. Cyclophilin D (CyPD) associates with p53 to induce apoptosis (121). Overexpression of miRNA-1281 which targets CyPD, protects human macrophages from *M. tuberculosis*-induced programmed necrosis and apoptosis (122). The nuclear body protein, Sp110 regulates miR-125a, miR-146a, miR-155, miR-21a and miR-99b expression in *M. tuberculosis*-infected RAW264.7 (123). It upregulates Bcl2 modifying factor (Bmf) by inhibiting miR-125a, thereby enhancing apoptosis. In primary human macrophages infected with *M. tuberculosis*, miR-579 induction downregulates its targets sirtuin 1 (SIRT1) and phosphoinositide-dependent protein kinase 1 (PDK1) and leads to macrophage apoptosis (124). miR-325-3p directly targets ligand of numb-protein X 1 (LNX1) (125), an E3 ubiquitin ligase of NIMA related kinase 6 (NEK6) which promotes NEK6 proteasomal degradation. The accumulation of NEK6 activates signal transducer and activator of transcription 3 (STAT3) signaling and inhibits apoptosis. On the other hand, Wu et al. (126) have shown that miR-21 is increased in alveolar macrophages of BCG-vaccinated mice, and miR-21 enhances apoptosis of bone marrow derived dendritic cells (BMDCs) from BCG-vaccinated mice most likely by targeting Bcl-2. 6 kDa early secretory antigenic target (ESAT-6)-driven miR-155 targets suppressor of cytokine signaling 1 (SOCS1) to augment macrophage apoptosis by elevating caspase 3 activity (127). TLR-2/MyD88/NF-κB dependent induction of miR-27b suppresses NF-κB (128). Further, miR-27b targets Bcl-2-associated athanogene 2 (Bag2) to regulate apoptosis. miR-27b increases p53-dependent apoptosis to lower bacterial burden. Induction of let-7b-5p in *M. tuberculosis* infected THP-1 macrophages leads to reduction in its target Fas, and inhibition of apoptosis (29). Activated Akt phosphorylates and inhibits the transcriptional activity of forkhead box O3 (FOXO3) (129). Upon Akt inactivation, dephosphorylated FOXO3 translocates to the nucleus where it activates transcription of multiple pro‐apoptotic genes harboring the forkhead response elements (FRE) in their promoter regions (130). miR-223 targets FOXO3 (131). The upregulation of miR-223 during infection, therefore, likely plays a role in the attenuation of macrophage apoptosis. A list of miRNAs regulating apoptosis during *M. tuberculosis* infection is given in **Table 2**, and their roles are schematically presented in **Figure 2**. Manipulation of apoptosis by administration of miRNA agonists or antagonists deserves further exploration as a means of containing *M. tuberculosis in vivo*.

**Table 2:** MicroRNAs involved in the regulation of apoptosis during *M. tuberculosis* infection.

MicroRNA	Target	Reference
miR-99b	Tnf, Tnfrsf45	(59)
miR-20a-5p	Jnk2	(119)
miR-20b-5p	Mcl-1	(120)
miR-21-5p	Bcl-2	(45)
miR-1281	Cyclophilin D	(121)
miR-125a	Bmf	(123)
miR-579	Sirt1, Pdk	(124)
miR-155	Socs1	(127)
miR-27b	Bag2	(128)
miR-223	Foxo3	(131)
Let-7b-5p	Fas	(132)
miR-325-3p	Lnx1	(125)

## The Role of Long Non-Coding RNAs in Regulating the Immune Response to *M. tuberculosis*

The majority of lncRNAs are transcribed by RNA polymerase II (RNAPII) (133), although there are some that are transcribed by RNA polymerase III (RNAPIII) including the 7SL RNA genes (134). LncRNAs undergo transcriptional editing such as splicing and polyadenylation before finally adopting a stable structure. They are expressed in a manner that is dependent on cell and tissue types, the stage of development, and association with disease (10, 135). They show poor evolutionary conservation (136), although a small number of lncRNAs are well conserved (137). LncRNAs fall into different categories. Antisense lncRNAs are transcribed across the exons of protein coding genes, but from the opposite strand. The transcripts for long intergenic non-coding RNAs (lincRNAs) are located between protein coding genes. Enhancer RNA (eRNA) transcripts are bidirectionally expressed at active enhancer regions of the genome. These are mainly cis-acting and control interactions between promoters and enhancers to regulate gene expression. Intronic lncRNAs are transcribed from the introns of annotated protein coding genes. circRNAs interact with and interfere with the functions of miRNAs.

LncRNAs perform diverse functions among which is the regulation of immunity and host-pathogen interactions (138–140). They interact with other molecules through base pairing or secondary structures (141). Nuclear lncRNAs interact with chromatin remodelers to regulate the expression of neighboring or distal genes. Cytoplasmic lncRNAs interfere with stability and translation of mRNA and signaling pathways (142). They also interact with a range of other molecules. In summary, they can act as protein scaffolds, regulators of transcription, antisense RNA or miRNA sponges (143–147). LncRNAs are now acknowledged as major players in maintaining homeostasis and in disease (148, 149). Transcriptomic data have shown the differential expression of hundreds of lncRNAs during PRR stimulation (150–153). Several of these lncRNAs are trans‐acting regulators of protein‐coding genes such as TNF and HNRNPL related immunoregulatory long non-coding RNAc(THRIL) which regulates TNF‐α expression by forming a complex with the ribonucleoprotein (RNP) hnRNPL that acts at the TNF‐α promoter (154), or the lincRNA‐COX2 which regulates inflammatory gene expression in LPS‐stimulated bone marrow‐derived dendritic cells by interacting with hnRNP‐A/B and hnRNP‐A2/B1 (151) or the lincRNA-EPS which associates with chromatin and interacts with hnRNPL to repress immune-responsive gene-1 (IRG) expression (150).

LncRNA expression profiles have been documented by transcriptome analysis from pulmonary TB (PTB) patients *vs*. healthy individuals (155). PTB patients showed differential expression of 449 lncRNAs. The expression profiles of lncRNAs were investigated by transcriptome sequencing. Lnc-HNRNPU-1:7 and lnc-FAM76B-4:1 were the most upregulated and downregulated lncRNAs respectively, in PTB patients compared to healthy individuals. In separate studies (156), a comprehensive analysis has shown that compared to healthy volunteers, 1,429 and 2,040 lncRNAs are deregulated in the PBMCs from MDR-TB and drug-sensitive TB patients, respectively. However, these results have not been analyzed with respect to their relevance and implications in the context of infection. During TB infection, the expression of the lnc-TGS1–1 target miR-143, is increased due to elimination of the sponge effect of lnc-TGS1-1. This likely suppresses downstream innate immune signaling (157). Along similar lines, removal of the inhibitory effect of lnc-AC145676.2.1–6 results in upregulation of miR-29a and interference with the TLR pathway (158).

LncRNA nuclear paraspeckle assembly transcript 1 (NEAT1) is expressed at higher levels in PBMCs from patients with tuberculosis than in healthy individuals, and in infected THP1 cells compared to uninfected ones (159). Knockdown of NEAT1 increases bacterial CFUs in infected THP1 cells, suggesting that NEAT1 is required for mounting a bactericidal response against *M. tuberculosis*. Comprehensive expression profiles of murine RAW264.7 macrophages infected with *M. tuberculosis* showed differential regulation of 1,487 lncRNAs (791 up and 696 down) (160). LncRNA profiles have also been generated for human macrophages infected with *M*. *tuberculosis* (161). In a study involving 473 healthy individuals and 467 TB patients, lnc-AC145676.2.1-6 and lnc-TGS1-1 expression levels were lower in TB patients. LincRNA cyclooxygenase 2 (Cox2) is activated by the TLR signaling pathway in macrophages and mediates both activation and repression of genes (151). LincRNACox2 is increased in macrophages infected with *M. tuberculosis* (15). Knockdown of lincRNA Cox2 decreased NF-κB and STAT3 while increasing apoptosis in infected macrophages. LncRNA HOX Transcript Antisense RNA (HOTAIR) targets the H3K27 methylase complex polycomb repressive complex 2 (PRC2) to distinct loci to regulate H3K27 methylation at these loci (162). Subuddhi et al. (163) have shown that lncRNA HOTAIR is downregulated in *M. tuberculosis* H37Rv-infected THP-1 macrophages, but upregulated in *M. tuberculosis* H37Ra-infected macrophages, leading to differential association of H3K27 methylation marks at the dual specificity phosphatase 4 (DUSP4) and SATB homeobox 1 (SATB1) loci. Downregulation of HOTAIR, facilitated increased transcription of DUSP4 and SATB1, both of which favored the survival of *M. tuberculosis* H37Rv in macrophages. Li et al. (164) showed a significant reduction in PC-esterase domain containing 1B antisense RNA1 (PCED1B-AS1) expression in monocytes from patients with active TB compared with that in monocytes from healthy individuals. Suppression of PCED1B-AS1 in infected macrophages significantly attenuated TNF-α-induced apoptosis with decreased cleaved caspase-3 and a concomitant increase in Bcl-2. Further, PCED1B-AS1 serves a miR-155 sponge. Its knockdown leads to upregulation of miR-155 and inhibition of its targets RHEB and FOXO3, culminating in enhanced autophagy (107, 165). Sun et al. (166) have reported that LncRNA maternally expressed 3 (MEG3) is activated by vitamin D and plays a regulatory role in carcinogenesis (167). It is highly expressed in pulmonary tuberculosis (PTB) (166). Lnc-MEG3 binds miR-145-5p (168). The relationship between Lnc-MEG3, miR-145-5p and apoptosis has been strengthened by infecting RAW264.7 with *M. bovis* BCG under conditions of overexpression or knock down of MEG3 (166). The authors have shown that inhibition of Lnc-MEG3 is associated with increased miR-145-5p and decreased apoptosis. Lnc-MEG3 also induces autophagy in macrophages infected with *M. bovis* BCG (16).

LncRNAs regulate the response of T and B cells to *M. tuberculosis* by regulating chromatin modification states. During *M. tuberculosis* infection, CD244, a T cell-inhibitory molecule, mediates inhibition of IFN-γ and TNF-α expression by inducing lncRNA-CD244 which interacts with the H3K27 methylase enhancer of zeste homolog 2 (EZH2), and mediates modification of a more repressive chromatin state at the *infg* and tnfa loci (169). Knock down of lncRNA-CD244 significantly enhances IFN-γ and TNF-α expression and improves protective immunity of CD8+ T cells.

**Figure 3:**
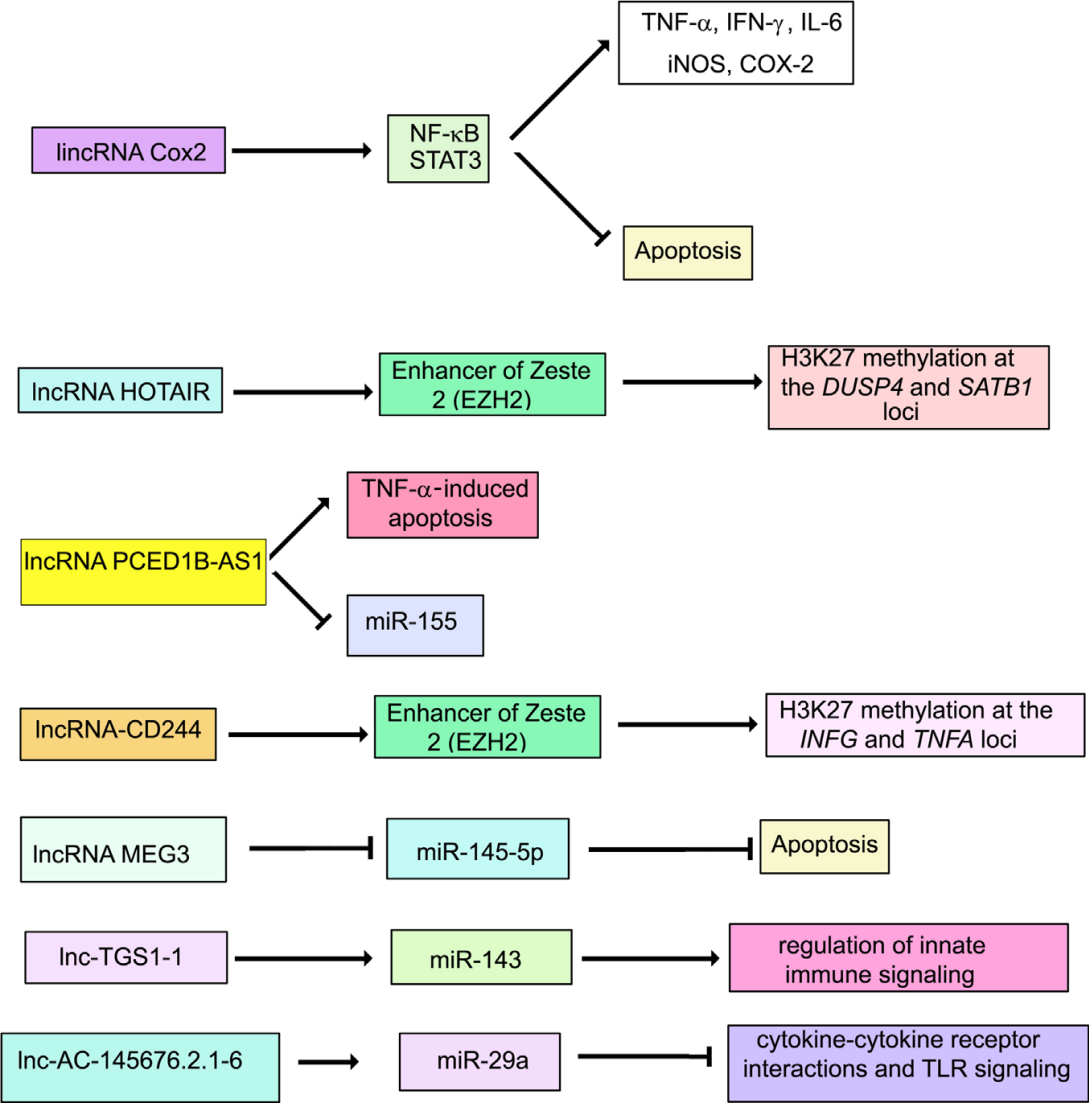
Schematic representation of the role of lncRNAs during *M. tuberculosis* infection.

Differentially expressed lncRNAs have been identified in CD4+ T cells in latent TB (170). Fu et al. (171) have reported that 844 lncRNAs were differentially expressed in B cells from individuals with TB. Concomitant with the dysregulation of several lncRNAs, adjacent protein-coding genes were also deregulated. For example, SOCS3 and its neighboring lncRNA XLOC_012582 were highly expressed in B cells from TB patients. The relevance of this observation in the context of TB, awaits further investigation. LncRNAs that regulate host signaling pathways during *M. tuberculosis* infection, are shown in **Figure 3**.

## Non-Coding RNAs as Markers of Active TB and LTBI

The potential use of miRNAs in the diagnosis of tuberculosis has been reviewed by Sabir et al. (172). In particular, the role of exosomal miRNAs as biomarkers of TB is of interest (173). MiRNAs miR-20a, miR-20b, miR-26a, miR-106a, miR-191, miR-486 are differentially expressed in exosomes from TB compared to healthy individuals (174). The overexpressed miRNAs showed reduction in expression after two months of anti-tuberculosis therapy. In a separate study, miR-484, miR-425, and miR-96-3-p were reported to be enriched in exosomes from patients with active TB (175). Some studies have specifically explored the potential of exosomal miRNAs as biomarkers of LTBI. Small RNA sequencing of serum exosomes from LTBI and TB patients showed that let-7e-5p, let-7d-5p, miR-450a-5p and miR-140-5p were specifically expressed in LTBI, whereas miR-1246, miR-2110, miR-370-3p, miR-28-3p and miR-193-5p, were specifically associated with active TB (176). In a separate study, miR-122-5p expression was observed to be significantly higher in the exosomes of LTBI than in active TB (177). On the other hand, let-7i-5p, miR-148a-3p, miR-21-5p and miR-423-5p showed higher expression in active TB than in LTBI. Independent miRNA profiling from PBMCs or serum suggest that a group of differentially regulated miRNAs distinguish active TB from latent TB (178, 179). miR-889 expression was increased in patients with latent TB compared to uninfected individuals (98). Latorre et al. (180) have identified whole blood-derived miRNA signatures that enable distinguishing active TB from latent TB. A systems biology approach has been adopted by Lin et al. (181) to generate a unique miRNA-gene regulatory network for LTBI by analyzing numerous microarray data sets. The miRNAs in this network were identified with the Hippo signaling pathway (miR-212-3p, miR-29a-5p, miR-29b-3p), ECM–receptor interaction (miR-432-5p, miR-148b-5p, miR-29b-3p, miR-532-5p), and the PI3K-Akt signaling pathway (miR-29b-3p). The reciprocal relationship between MAPK1 and miR-212-3p was validated. The authors suggest that the interaction of miR-212-3p with MAPK1 could regulate the PI3K-Akt signaling pathway to affect the transmission of *M. tuberculosis*.

In a similar vein, attempts have been made to use lncRNA expression as a means of TB diagnosis. Fang et al. (182) have analyzed the GEO dataset (GSE94907) and identified the differentially regulated lncRNAs NONHSAT101518.2, NONHSAT067134.2, NONHSAT148822.1 and NONHSAT078957.2 which are downregulated in the plasma of active TB patient plasma compared with the healthy individuals, as potential biomarkers. In a separate study, a total of 163 upregulated lncRNAs and 348 downregulated lncRNAs were identified in the plasma of PTB patients (18). Four differentially expressed lncRNAs, NR_038221, NR_003142, ENST00000570366 and ENST00000422183 were confirmed by qRT-PCR. The potential target genes of NR_003142 in immune pathways were TLR6, nucleotide binding oligomerization domain containing 2 (NOD2), HLA-DQB and IL6ST suggesting that it could influence TB pathology. Hu et al. (183) have shown that three lncRNAs, ENST00000497872, n333737, and n335265 are differentially expressed in blood samples of active TB patients compared to healthy individuals and suggested that these may serve as potential biomarkers for clinically diagnosed PTB patients. Of these, lncRNA ENST00000497872 is located close to the immune related gene immunoglobulin heavy constant alfa 1 (IGHA1) and n333737 is located close to the immune related gene T cell receptor alfa variable 1-2 (TRAV1-2). Lnc-TGS1–1 and lnc-AC145676.2.1–6 are downregulated in TB patients (184). In separate studies, 41 lncRNAs have been reported to be deregulated in a comparison between healthy subjects, active TB and LTBI (170). These studies suggest that targeting miRNAs and lncRNAs for development of HDTs, and as biomarkers of TB, deserve detailed investigation.

## Concluding Remarks

This review outlines the role of miRNAs and lncRNAs in regulating functions in myeloid cells that are critical in determining the outcome of *M. tuberculosis* infection such as inflammation, macrophage polarization, metabolism, autophagy and apoptosis. In spite of the extensive literature presently available, there are several roadblocks and contradictions along the path towards understanding the role of miRNAs in human tuberculosis, arising out of differences between animal infections and human disease and variations in methodology (such as strains used, multiplicity of infection, and time of infection) between different studies. One such example is the role of miR-21 in infection. Mir-21, on the one hand, inhibits glycolysis and the production of bactericidal effectors such as ROS and NO (67), providing a permissive milieu for the growth of *M. tuberculosis*. On the other hand, Zhao et al. (45) report that miR-21 targets Bcl-2, suggesting that it accelerates apoptosis. This would deprive *M. tuberculosis* of its niche within the host. As of now, there are no *in vivo* studies to resolve such contradictions on the actual role of miR-21 in infection.

Methods for rapid diagnosis of TB are urgently required. miRNAs have been explored for the diagnosis of a range of diseases such as cancer. Several studies have tested the potential of miRNAs as diagnostic markers for TB. Among the miRNAs that have been discussed here, at least four have been consistently associated with TB namely miR-144-3p, miR-144-5p, miR-146a and miR-155. miR-144-3p levels are higher in sputum and serum from patients with active TB compared to healthy uninfected individuals (185). miR-144-3p targets ATG4a to inhibit autophagy during *M. bovis* BCG infection. miR-144-5p levels are increased in PBMCs from active TB patients compared to healthy individuals (186). miR-144-5p targets DRAM2 to regulate autophagy. Alveolar macrophages from smear-positive patients show lower levels of miR-146a than those from smear-negative patients or healthy uninfected individuals (187). miR-146a targets IRAK1 and TRAF6. miR-155 levels are reportedly reduced in serum from patients with active TB (188), and increased after TB therapy. miR-155 has been reported to regulate inflammatory cytokines, autophagy and apoptosis, during infection of macrophages. miR-196b and miR-376c have been proposed as serum markers of active TB and LTBI (179). The differential expression of miRNAs in body fluids such as blood and sputum, distinguishes TB patients from healthy individuals, or LTBI; treated from untreated patients; and those infected with hypervirulent or drug-resistant strains (189). Serum exosomes have also been evaluated for the presence of miRNA biomarkers capable of distinguishing latent TB, active TB and healthy individuals. A recent study has suggested the differential expression of miR-625-3p in urine samples, as a marker for TB diagnosis (190). This highlights the pivotal role of miRNAs in TB. However, as of now miRNA signatures characteristic of tuberculosis disease states, remain to be defined (191). In particular, detailed investigation needs to be done in terms of identifying biomarkers for latent TB. There are obvious pitfalls in comparing miRNA levels during *in vitro* and *in vivo* infection across different studies, arising out of difficulties in pinning down different stages of the disease, choice of platform for miRNA identification, choice of strain of *M. tuberculosis*, age and sex of individuals, methods of RNA extraction, and sample size, to mention a few. However, there is substantial literature that underscores the need to explore development of miRNA delivery vehicles and miRNA-based therapeutics as possible strategies for shortening the duration of therapy (9). Some studies in animal models have shown promise. Lou et al. (51) have demonstrated that intravenous administration of miR-20b mimics could attenuate the inflammatory response in a mouse model of infection by inhibiting activation of the inflammosome. In a *M. marinum* model, transfection of microglia with miR-124-3p promoted apoptosis through targeting of STAT3 to decrease mycobacterial proliferation (192). It is therefore important to devise efficient delivery methods for miRNAs that would protect them from circulating RNAse and target them to the site of infection. The available technologies for miRNA delivery include nanoparticles as vehicles, use of viral vectors, exosome-like vesicles and lipid-based delivery systems. A silica nanoparticle-based targeted delivery of miR-34a, to neuroblastoma tumors in a murine orthotopic xenograft model, has been demonstrated (193). It must be mentioned that nanoparticle based delivery platforms are associated with high cost of production, non-specific distribution and cytotoxicity. Administration of miR-26a in a mouse model of hepatocellular carcinoma using adeno-associated virus (AAV) inhibited cancer cell proliferation (194). However, viral delivery methods may elicit unwanted inflammatory responses. There are several reports that suggest that miRNA could probably be delivered to the lung for the treatment of TB. Mice treated with pre-miR-133b containing lipoplexes through the tail vein, showed 52-fold higher expression of miR-33b in the lungs compared to untreated mice (195). miRNA targeting drugs are in clinical trials for the treatment of hepatitis C virus infection *via* targeting miR-122, polycystic kidney disease *via* targeting miR-17, cutaneous T cell lymphoma *via* targeting miR-155 and keloids *via* targeting miR-19 (196). miRNA based therapeutic options need to be pursued in earnest for the management of TB.

The association of lncRNAs with tuberculosis, and their functions have been less extensively explored than the role of miRNAs. Much more work needs to carried out, before we understand their link to disease outcomes.

## Author Contributions

JB and MK reviewed the literature and wrote the manuscript. All authors contributed to the article and approved the submitted version.

## Funding

JB is supported by funding from the J.C. Bose Fellowship (SB/S2/JCB-049/2016). MK is supported by the Council for Scientific and Industrial Research, Emeritus Scientist Scheme (21(1088)/19/EMR-II).

## Conflict of Interest

The authors declare that the research was conducted in the absence of any commercial or financial relationships that could be construed as a potential conflict of interest.
